# Mitochondrial DNA Variation Contributes to the Aptitude for Dressage and Show Jumping Ability in the Holstein Horse Breed

**DOI:** 10.3390/ani12060704

**Published:** 2022-03-11

**Authors:** Laura Engel, Doreen Becker, Thomas Nissen, Ingolf Russ, Georg Thaller, Nina Krattenmacher

**Affiliations:** 1 Institute of Animal Breeding and Husbandry, Christian-Albrechts-University, 24098 Kiel, Germany; gthaller@tierzucht.uni-kiel.de (G.T.); nkrattenmacher@tierzucht.uni-kiel.de (N.K.); 2 Institute of Genome Biology, Research Institute for Farm Animal Biology (FBN), 18196 Dummerstorf, Germany; becker.doreen@fbn-dummerstorf.de; 3 Verband der Züchter des Holsteiner Pferdes e.V., 24106 Kiel, Germany; nissen@holsteiner-verband.de; 4 Tierzuchtforschung e.V. München, 85586 Grub, Germany; ingolf.russ@tzfgen-bayern.de

**Keywords:** mitochondrial DNA, mitochondrial association analysis, Holstein horse, maternal lineages, show jumping, dressage

## Abstract

**Simple Summary:**

In the Holstein horse breed, maternal lineages are considered to be of major importance for the breeding success and can be examined through analysis of the maternally inherited mitochondrial DNA (mtDNA). Since mitochondrial genes are involved in energy metabolism, variation might contribute to differences in performance characteristics, as has already been pointed out in humans and racehorses with respect to endurance. No corresponding studies have yet been conducted for the athletic performance of warmblood breeds and, thus, the aim of this study was to investigate the influence of mitochondrial variation on the performance of Holstein mares. The data set used for this study was composed of both sequenced and non-sequenced mares of 75 maternal lineages as a previous study revealed that Holstein mares within a maternal lineage had identical mtDNA haplotypes regarding their non-synonymous variants. Association analyses were performed using estimated breeding values (EBVs) based on information from sport and breeding events. We observed mitochondrial single nucleotide polymorphisms (SNPs) significantly associated with one or more of the examined EBVs and identified mitochondrial haplogroups with a particular aptitude for dressage or show jumping.

**Abstract:**

Maternal lineages are considered an important factor in breeding. Mitochondrial DNA (mtDNA) is maternally inherited and plays an important role in energy metabolism. It has already been associated with energy consumption and performances, e.g., stamina in humans and racehorses. For now, corresponding studies are lacking for sport performance of warmblood breeds. MtDNA sequences were available for 271 Holstein mares from 75 maternal lineages. As all mares within a lineage showed identical haplotypes regarding the non-synonymous variants, we expanded our data set by also including non-sequenced mares and assigning them to the lineage-specific haplotype. This sample consisting of 6334 to 16,447 mares was used to perform mitochondrial association analyses using breeding values (EBVs) estimated on behalf of the Fédération Équestre Nationale (FN) and on behalf of the Holstein Breeding Association (HOL). The association analyses revealed 20 mitochondrial SNPs (mtSNPs) significantly associated with FN-EBVs and partly overlapping 20 mtSNPs associated with HOL-EBVs. The results indicated that mtDNA contributes to performance differences between maternal lineages. Certain mitochondrial haplogroups were associated with special talents for dressage or show jumping. The findings encourage to set up innovative genetic evaluation models that also consider information on maternal lineages.

## 1. Introduction

Since the middle of the 20th century, the Holstein horse has been intensively bred for its athletic performance and aptitude for show jumping, eventing, or dressage, with an explicit focus on show jumping ability. The World Breeding Federation for Sport Horses (WBFSH) publishes annually the most successful studbooks in the above-mentioned disciplines based on competition results. In both show jumping and eventing, the Holstein horse has been ranked among the top 10 breeds over the past 10 years, and even in dressage, a ranking in the top third has consistently been achieved [1]. Holstein horse breeders rely on maternal lineages, which are considered to contribute substantially to the breeding success, and their documentation dates back to the beginning of the 19th century. Mares with unknown parents were defined as founder mares resulting in more than 8900 different maternal lineages up to today. Due to World War II and the growing mechanization of agriculture, the number of Holstein mares decreased markedly, accompanied by a loss of maternal lineages. Furthermore, the focus of selection has shifted from the use for agriculture, carriage, and cavalry toward athletic performance. To achieve this goal, English Thoroughbred and Anglo-Norman stallions were increasingly used for breeding, while the studbook remained closed. Today, 437 maternal lineages exist, comprising 5412 active brood mares [2].

The impact of maternal lineages on breeding success can be examined through analysis of the mitochondrial DNA (mtDNA) since it is inherited maternally without recombination. The equine circular mitochondrial genome is 16,660 bp in length and comprises 37 genes, 13 of which encode for respiratory chain proteins involved in energy metabolism in addition to two ribosomal and 22 transfer RNAs essential for protein synthesis. The mitochondrial genome harbors candidate genes, variants of which may possibly influence adenosine triphosphate (ATP) synthesis during exercise [3].

The performances of the Holstein horse, e.g., the upward movement during jumping, are highly energy demanding, relying particularly on aerobic capacity [4]. Since aerobic energy production takes place in mitochondria, it is reasonable to assume that variation in mitochondrial genes could possibly have an effect on athletic performance. Therefore, we recently analyzed mtDNA sequences from 271 Holstein mares belonging to 75 maternal lineages [5]. We were able to show that considerable molecular variation among mitochondrial genomes of different maternal lineages exists in Holstein horses and identified a total of 78 haplotypes that could be assigned to eight distinct haplogroups. The mtDNA sequences of mares within a haplogroup showed high levels of similarity. Within a lineage, identical mtDNA haplotypes were found in all mares with respect to the non-synonymous substitutions [5], which is in accordance with results from studies in other breeds [6,7]. Based upon these findings, we enlarged our sample size by including both sequenced and non-sequenced mares belonging to one of the maternal lineages analyzed in our previous study [5], assuming that the non-sequenced mares have the same mitochondrial haplotype of non-synonymous variants as the sequenced mares of the same lineage. Using the resulting sample, we investigated the involvement of mitochondrial variants with regard to the performance of Holstein mares. Estimated breeding values (EBVs) were used as targets to perform mitochondrial association analyses. For the Holstein horse, two separate genetic evaluation systems were run. On the one hand, EBVs were estimated population-specific on behalf of the Holstein Breeding Association (HOL) for traits recorded at studbook inspection and mare performance test. On the other hand, EBVs were estimated across all German warmblood breeds on behalf of the Fédération Équestre Nationale (FN) based on the results from sport and breeding events. A detailed description of the breeding value estimation can be found in the work of [5,8,9] Supplementary File S1.

## 2. Materials and Methods

For this study, a representative sample of Holstein mares belonging to 75 lineages was used to perform an association study between mitochondrial variants and EBVs estimated on behalf of the FN (FN-EBVs) and on behalf of the Holstein Breeding Association (HOL-EBVs).

### 2.1. Sampling, Sequencing, and Enlargement of the Data Set

Initially, 493 mares with extensive phenotypes, i.e., mares that preferably have information from studbook inspection, mare performance test, and from sport events, were preselected, and the respective breeders were asked to collect hair samples during routine care, e.g., combing the mane or tail. Besides this, mares were selected based on the availability of genotypes that were provided by an in-house project to allow consideration of interactions between the mitochondrial and the nuclear genome in upcoming studies. For the in-house project, the maternal lineage was of no interest, and mares showed a low level of preselection and a low pedigree relationship. After two sampling periods in 2019, hair samples were available for a total of 271 mares, which were sent in by 207 breeders.

For each sample, DNA was extracted from 20 to 25 hair roots using a modified protocol according to the work of [10]. The PRIMER 3 software (Version 4.1.0, https://primer3.ut.ee/, accessed on 10 January 2022) was used to create primer pairs for amplification of the mitochondrial genome [11]. Polymerase chain reaction (PCR) amplifications were performed in a 12 µL reaction volume including 20 ng total DNA, 0.2 µM of the forward and reverse primer, 200 µM deoxyribonucleoside triphosphates (dNTPs), 1.25 U of the PrimeSTAR GXL DNA-Polymerase (Takara Bio, Shiga, Japan), and the corresponding reaction buffer. The sequencing was performed in 36 reactions using the ABI 3130xl Genetic Analyzer and the BigDye^®^ Terminator v3.1 Cycle Sequencing Kit (Applied Biosystems, Foster City, CA, USA). A more detailed description of the methods can be found in the work of [5].

Analysis of the mtDNA sequences was performed using the software Sequencher 5.0 (Gene Codes Corporation, Ann Arbor, MI, USA). If the sequences were ambiguous, the sequencing was repeated, and, if necessary, these samples were excluded from the analysis. For all samples, the repetitive part of the non-coding region was excluded due to failure in sequencing. The sequences were compared to the GenBank reference sequence X79547.1 the sequences of the mitochondrial haplotypes are provided in Supplementary File S2. 

A total of 467 polymorphic sites were found, but only the 101 non-synonymous substitutions previously reported in the work of [5] were considered for the evaluation.

The sample was extended with non-sequenced mares belonging to the 75 lineages that were previously analyzed [5]. This was done based on the finding that all mares belonging to the same lineage have identical haplotypes regarding the non-synonymous mitochondrial substitutions. For 2020, HOL-EBVs for studbook inspection (SBI) and mare performance test (MPT) were estimated for 17,745 and 8167 mares, respectively. FN-EBVs were available for 13,920 mares. The mares were born between 1944 and 2017 and assigned to maternal lineages, which made up 17.4% of all lineages of the current breeding population and to which 56.4% of all active broodmares can be assigned. The number of mares per lineage in the current data sets ranges from 16 to 998 for the FN-EBVs, 28 to 1248 for the EBVs for studbook inspection (SBI-EBVs), and from five to 520 for the EBVs for mare performance test (MPT-EBVs). In both genetic evaluation systems, the majority of lineages was represented by 50 to 200 mares. Based upon previous results from the work of [5], the lineages can be assigned to eight different haplogroups defined by the authors of [12]. Table 1 shows the number of lineages, the number of sequenced mares, and the total number of mares per haplogroup in this sample.

### 2.2. Phenotypic Data

HOL-EBVs and FN-EBVs were used as targets for this study. All breeding values were routinely standardized with a mean of 100 and a standard deviation of 20 points by FN and HOL. For this study, only breeding values with a reliability ≥30% were used, leading to a loss of 1298, 1833, and 949 mares with breeding values for SBI, MPT, and FN, respectively. Reliabilities for HOL-EBVs were provided summarized for all SBI and MPT traits, respectively. Heritability estimates for all traits were moderate, except for the traits forehand and hindquarters that show low heritability estimates.

### 2.3. Data Analysis

Association testing was performed in PLINK version 1.9 software [13] using the ASSOC function. We excluded 50 mtSNPs that did not reach the minor allele frequency (MAF) of more than 1%, thus, leaving 51 mtSNPs for the association analysis. The mtSNPs were named according to their position (bp) on the mitochondrial genome as they have not been previously reported in other breeds. The Bonferroni-corrected significance threshold was *p* < 5 × 10^−4,^ but we defined a significance threshold of *p* < 5 × 10^−8^ to avoid too many false positive results. The pairwise linkage disequilibrium (LD) was calculated as the correlation between mtSNP pairs (r^2^) using the Haploview software [14]. Values of r^2^ = 1 and r^2^ = 0 are illustrated in black and white, respectively, with different shades of gray for the intermediate values. For mtSNPs in perfect LD (r^2^ = 1), only one mtSNP was stated in all further evaluations. The results were visualized with the software Synthesis-View [15]. Further statistical analyses were performed using R version 4.0 [16].

## 3. Results

A sample of 5302–12,971 mares with FN-EBVs, 16,447 mares with SBI-EBVs, and 6334 mares with MPT-EBVs was used, where only EBVs with a reliability above 30% were considered. Table 2 provides an overview of the complete data set used for the analyses and the descriptive statistics for EBVs and reliabilities, respectively. The average EBV of the genetic evaluation of the FN ranges between 82.26 (±12.01) for the trait “sport and breeding tests for young horses” (JPf) dressage and 109.37 (±18.95) for the trait “highest level in competition” HEK jumping. There are differences between the average EBVs of the different disciplines: while average EBVs for dressage traits, including basic gaits and rideability, range between 82.26 (±12.01) and 89.95 (±12.35), those for show jumping are always above 100, with one exception for the trait ABP jumping (99.09 ± 13.87). For SBI, average EBVs range between 90.82 (±22.45) and 95.34 (±19.88) for the traits HOL hindquarters and HOL type, respectively. The average EBV for MPT range from 85.10 (±22.14) for HOL free jumping to 98.81 (±23.54) for HOL walk.

**Figure 1:**
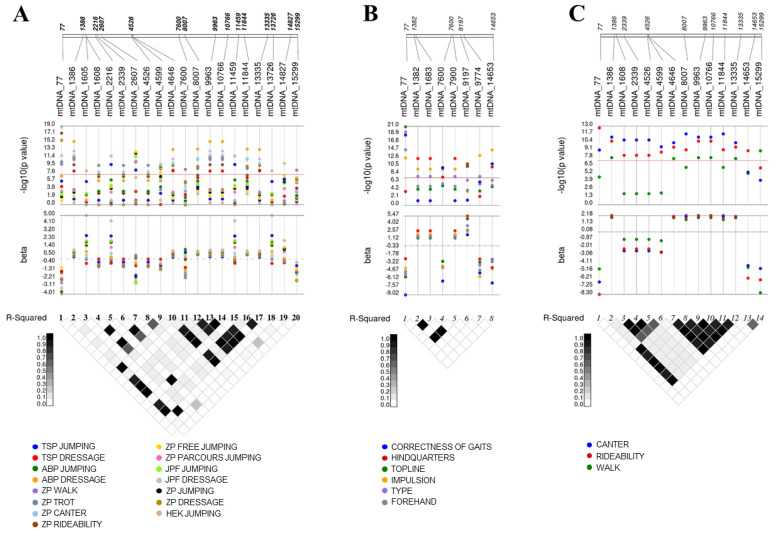
Mitochondrial SNPs significantly associated with breeding values. Association analyses were performed to identify mtSNPs associated with (**A**) FN breeding values, (**B**) breeding values for studbook inspection, and (**C**) breeding values for mare performance test. Only mtSNPs with *p* < 5 × 10^−8^ (indicated by the red line) are illustrated and are plotted according to their position on the mitochondrial genome. The values for the different traits are shown in different colors. LD between mtSNPs was measured as r^2^ using Haploview software.

20 mtSNPs were significantly associated with FN-EBVs. Associations were found for all traits except for HEK dressage. The name and position of the significant SNPs on the mitochondrial genome, as well as the −log_10_ (*p*-values) and the estimated effect sizes, are shown in Figure 1, where the values for the different traits are indicated in different colors. The red line specifies the significance threshold of *p* < 5 × 10^−8^. Additionally, r^2^ between mtSNP pairs is shown. Only significant mtSNPs with *p* < 5 × 10^−8^ are depicted.

A total of eight mtSNPs were associated with the SBI-EBVs. Two of the significant mtSNPs were also associated with FN-EBVs. The other six are exclusively significant for SBI-EBVs. For MPT, 14 mtSNPs are significantly associated with the EBVs walk, canter, and rideability. Thirteen mtSNPs of them are also significant for FN-EBVs.

Comparisons of the average EBVs of the two alleles for each significant mtSNP were performed for each trait. The results for the association between FN-EBVs and mtSNPs are shown in Table A1. It further indicates the percentage of maternal lineages in a haplogroup carrying the minor allele. For example, the minor allele of the mtSNP mtDNA_2216 occurs in all four maternal lineages of haplogroup D, and these mares have an average EBV for the trait TSP jumping that is 5.29 points higher compared to the remaining mares studied. All maternal lineages of haplogroup B carry the minor alleles of two mtSNPs that are associated with higher EBVs in all dressage traits. All maternal lineages of haplogroup D have higher EBVs in six out of eight dressage traits and additionally in TSP jumping and HEK jumping. In haplogroup L, two sub-haplogroups consisting of three and one maternal lineages, respectively, show significant differences compared to the remaining sample. One sub-haplogroup shows lower EBVs in all jumping traits except HEK jumping, while the second sub-haplogroup has lower EBVs in six dressage traits. All maternal lineages of haplogroup N are characterized by reduced EBVs in trot and canter. Additionally, about three-quarters of them also show lower EBVs in four dressage traits and in free jumping. There is one mtSNPs whose minor allele occurs in 13 maternal lineages from five different haplogroups, and that is associated with higher EBVs in HEK jumping.

The results from the association analysis between SBI-EBVs and mtSNPs are shown in Table A2. All maternal lineages of haplogroup P show higher breeding values in HOL type, HOL hindquarters, and HOL impulsion. A sub-haplogroup of haplogroup G carries the minor allele of one mtSNP is associated with lower EBVs in HOL hindquarters, HOL correctness of gaits, and HOL impulsion. Besides this, only three single maternal lineages from three different haplogroups have significantly different EBVs in the six SBI traits.

Table A2 also provides the results from the association analysis between MPT-EBVs and mtSNPs. Significant associations could be found for the three traits HOL canter, HOL walk, and HOL rideability, with all maternal lineages from haplogroup B showing higher EBVs for these traits. For HOL canter and HOL rideability, all maternal lineages from haplogroup B have higher EBVs, while all maternal lineages from haplogroup N have lower EBVs compared to the remaining sample.

## 4. Discussion

This study presents the first association analysis between mitochondrial variants and EBVs estimated for a show jumping breed and revealed that the mtDNA and, thus, maternal lineages were significantly associated with sport performance. A representative sample of Holstein mares was used, of which 1.51%, 1.62%, and 1.41% of the mares for SBI, MPT, and FN, respectively, were sequenced. As mitochondrial haplotypes were found to be highly consistent within maternal lineages [5], haplotypes of their respective maternal lineage were assigned to non-sequenced mares. Pedigree errors, and thus, incorrect assignment into maternal lineages, cannot be completely ruled out, but our previous study has shown that documentation of pedigrees in the Holstein breed is very accurate [5]. FN-EBVs and HOL-EBVs were used as phenotypes, although the use of EBVs in association studies remains controversial due to presumed high false discovery rates [17]. To reduce the risk of false positive results and, nevertheless, obtain reliable results, a large number of samples, ranging from 6334 to 16,447, and a stringent significance threshold that differs from the Bonferroni-corrected significance threshold was used.

### 4.1. Mitochondrial Variants

A total of 51 non-synonymous mtSNPs were used for the association analyses. A total of 24 different mtSNPs could be associated with the examined EBVs. As can be seen in Figure 1, many of them are highly correlated, which is not surprising since the mtDNA does not recombine. The number of associated mtSNP pairs and triplets in total LD is shown in Table 3. Additionally, it provides an overview of the mtSNPs used for the association analyses and the results, including the total number of significantly associated mtSNPs and the number of associated mtSNPs for HOL- and FN-EBVs. The associated mtSNPs are located in the 12 mitochondrial genes *s-rRNA*, *l-rRNA*, *ATP8*, *COX2*, *COX3, CYTB*, *ND2*, *ND3*, *ND4*, *ND4L*, *ND5,* and *ND6*. The *rRNA* genes are involved in mitochondrial protein synthesis, and the protein-coding genes encode for subunits of complex I, III, IV, and V of the mitochondrial respiratory chain [18].

Considering the FN-EBVs, the same mtSNPs are significant for the traits ZP trot and ZP canter, which is in accordance with the high genetic correlation (r_g_ = 0.69) of these traits [9]. ZP rideability also shows high concordance with the two traits with respect to the significant mtSNPs, and again, this is in line with the high positive genetic correlation of r_g_ = 0.67 for both ZP trot and ZP canter [9]. The EBVs for JPf dressage and ZP dressage are derived, among others, from the above-mentioned EBVs (Supplementary File S1, Table S1) and, predictably, show the same significant mtSNPs. It has already been reported that gaits and rideability are highly correlated with dressage traits in German warmblood breeds [19]. Furthermore, it can be observed that different mtSNPs are significant in the dressage and jumping traits. Noteworthy is, e.g., the variant mtDNA_2607, which is significantly associated with the six jumping traits (TSP jumping, ABP jumping, ZP free jumping, ZP parcours jumping, JPf jumping, and ZP jumping) but has no effect on the dressage, gait, and rideability traits. The minor allele is associated with lower EBVs in all jumping traits. Similar observations were made for the EBVs of MPT. The traits HOL canter and HOL rideability show high agreement in terms of significant SNPs, while two different mtSNPs are significant for the trait HOL walk. Additionally, a comparison between the genetic evaluations of the FN and the Holstein Breeding Association revealed that almost the same mtSNPs are significant for the traits walk, canter, and rideability, which was expected since the correlations between the FN-EBVs and HOL-EBVs for the gaits and rideability are high, ranging from 0.91 to 0.92. Nevertheless, EBVs for traits from MPT estimated on behalf of the FN and on behalf of HOL were used for the evaluations since HOL-EBVs are estimated population-specific, while FN-EBVs are estimated jointly for all German warmblood breeds. For the conformation traits HOL type, HOL topline, HOL forehand, and HOL hindquarters derived from SBI, six exclusive mtSNPs are significant that did not occur in other traits. When performing analogous analyses for other breeds, it is therefore recommended to consider population-specific EBVs besides FN-EBVs.

### 4.2. Mitochondrial Haplogroups and Performance

In a previous investigation, we could assign the maternal lineages to eight haplogroups, whereby all mares from one lineage belong to the same haplogroup. Within a haplogroup, the mtDNA sequences of mares are very similar [5]. Taking into account the previous and current results, some further conclusions can be drawn. In all maternal lineages of haplogroup B, variations occur that were associated with higher EBVs in the dressage traits of the FN genetic evaluation, as well as higher EBVs for the basic gaits and rideability. Consequently, a pronounced dressage ability could be attributed to the 24 lineages of this haplogroup. Haplogroup D shows partly the same variations as haplogroup B resulting in higher EBVs in the dressage, gait, and rideability traits. This is in accordance with previous results, where these two haplogroups show the lowest genetic differentiation in the overall comparison and thus, share large parts of their mtDNA sequence [5]. Additionally, the four lineages representing haplogroup D show higher EBVs for two jumping traits, and thus, this haplogroup could be characterized as more versatile. There are three sub-groups auf haplogroup L, each differing from the rest of the sample: one sub-group composed of one lineage shows lower EBVs in two dressage traits, as well as basic gaits, rideability, and five traits derived from SBI. Another sub-group of three lineages shows reduced EBVs for all jumping traits estimated by the FN, except for HEK jumping. This group seems to be less predestined for elite show jumping performance. In contrast, the third sub-group consisting of six lineages seems to be more successful in show jumping. Noteworthy, all lineages of haplogroup P seem to be particularly in line with the preferred breed type, combined with strong hindquarters and impulsion. All lineages of haplogroup N have comparatively low EBVs for trot, canter, and rideability. Additionally, 10 of the 13 lineages are characterized by lower EBVs regarding dressage and free jumping.

The overall comparison shows that there is no haplogroup that stands out for special jumping ability. It rather shows, especially regarding the trait HEK jumping, that only single maternal lineages or sub-groups show higher EBVs. This indicates that the Holstein population is on an equal level regarding its jumping ability with high mean breeding values for all jumping traits (Table 2), which is in line with the breeding goal of the Holstein breed with a strong focus on show jumping ability. Mean FN-EBVs for dressage traits were always below 100, even for the favorable allele. This is because FN-EBVs were estimated, including all German warmblood breeds, thus, including a couple of horse breeds from breeding associations that put a higher emphasis on dressage performance.

The impact of mitochondrial variation on performance has already been studied intensively in humans, where a total of 18 mitochondrial genes have been shown to be associated with fitness and performance phenotypes [20]. For example, the work of [21] found significant differences in the frequencies of two haplogroups in Finnish endurance and sprint athletes. Mitochondrial haplogroups are also associated with endurance performance in Spanish and Japanese humans [22,23]. In parallel, respective research in horses has mainly focused on racing performance. Thoroughbreds [24] identified haplotypes associated with racing performance at different distances, and [3] reported a mutation in the mitochondrial 16S rRNA gene associated with low racing performance. Corresponding studies are lacking for warmblood breeds. However, there are some studies that statistically examined the influence of maternal lineages on sport performance. Polish jumping breeds and their performance during the three-day Polish Championships for Young Horses were studied by the authors of [25]. The maternal impact, defined as the proportion of the total variance explained by the maternal additive genetic variance, was high for all traits (jumping style score, penalty score for each day, and overall rating), ranging from 0.11 to 0.39. In Holstein horses, the work of [26] investigated the effect of the maternal lineage on traits recorded during studbook inspection and mare performance test. Up to 0.9% of the phenotypic variation could be explained by the maternal lineage for traits recorded at studbook inspection. The strongest effect was found for hindquarters [26]. For the mare performance test, maternal lineages explained up to 2.1% of the variation with the highest value for the trait canter under the rider. In racehorses, there is a commercially genetic test available (Equinome Speed Gene Test; PlusVital, Dublin, Ireland) to predict the aptitude for racing performance based on nuclear variation. Accuracy of testing could be enlarged by considering also results from mitochondrial association studies. Furthermore, the results can be considered for the implementation of genomic selection in horses and the design of the SNP chip.

### 4.3. Limitations

When interpreting the results, it must be considered that only the mtDNA was examined. Since the majority of mitochondrial proteins are nuclear-encoded, interactions between the mitochondrial and nuclear genome are to be expected. However, the results are striking, as mitochondrial variations cause average EBVs that differ in 1.19 to 12.27 points, although the mitochondrial genome represents only a very small part of the total DNA. Furthermore, we were not able to adjust for close maternal relationships on the genomic level, where mares belonging to the same lineage or haplogroup will probably also show more similarities with regard to the nuclear genome [27]. Nevertheless, the mares in the enlarged data set were only selected based on their maternal lineage, where even distant maternal relatives share mtDNA independently from their genomic relationship. It should be noted, however, that sires are not evenly distributed across maternal lineages, which could have led to overestimation.

Since multiple mitochondria and multiple copies of the mtDNA exist in a cell, both original and mutant mtDNA molecules can co-occur [6]. This phenomenon, known as heteroplasmy, is specific for mtDNA and caused by de novo mutations occurring either in the germline or in the somatic tissues. Heteroplasmy could not be considered in this study because we used Sanger sequencing, which is not sensitive enough to detect it. Next-generation sequencing technologies with sufficient coverage would enable the detection of heteroplasmy even at low levels. Total mtDNA sequences could thus be sequenced with higher accuracy and should be applied for further investigations of mtDNA. However, the analysis of mtDNA using Sanger sequencing did not indicate the presence of heteroplasmy, as mainly unambiguous signals were found in the chromatograms. In case of ambiguous signals, sequencing of the respective sequence segment was repeated and confirmed the absence of heteroplasmy. However, the proportion of mutant mtDNA in a cell could possibly have an impact on the expression of a phenotype. Furthermore, there might be differences in the degree of heteroplasmy between tissues, which was not considered in this study because only hair samples were examined [28]. Analysis of mtDNA of tissues essential for energy supply could be insightful but would only be possible in horses for slaughter. However, not all horses are registered as animals for slaughter as this is accompanied by a restriction in drug administration. Thus, the sample would be less representative.

Due to the above-mentioned limitations and considering that upscaling from a low number of mares with mtSNP genotypic data to the whole population was performed, the interpretation of the results should be treated with caution. Although this study demonstrates the great potential of mitochondrial association studies and the importance of mitochondrial variation for performance traits, it also shows that more research is needed to simultaneously account for nuclear and mitochondrial relatedness and thus, preventing a misinterpretation of the results.

## 5. Conclusions

This study is the first study performing a mitochondrial association study in warmblood horses for sport performance traits. A representative sample of Holstein mares from 75 maternal lineages was used to which more than half of all active broodmares can be assigned. Mitochondrial variants in 12 different genes were shown to be associated with HOL-EBVs and FN-EBVs. The entire population shows a high level of jumping ability, however, with maternal lineages that stand out both positively and negatively. An enlargement of the sample with further maternal lineages is recommended since many mtSNPs have been excluded due to low MAF, and especially mtSNPs occurring in single sub-haplogroups have been found to be associated with the EBVs. Conclusively, the results provide evidence to revise the current genetic evaluation models by including information on maternal lineages. However, further work is needed to quantify the benefit of extended genetic evaluation models.

## Figures and Tables

**Table 1:** Overview of the data set and distribution of lineages among haplogroups.

Haplogroup ^a^	Number of Lineages	Number of Sequenced Mares	Number of Total Mares with ^b^		
			FN Breeding Values ^c^	SBI Breeding Values	MPT Breeding Values
B	24	72	3091	3952	1618
D	4	13	290	352	120
G	9	33	2099	2590	1057
I	5	14	817	1053	420
K	1	3	112	150	42
L	16	78	3646	4650	1716
N	13	11	2209	2770	1016
P	3	47	707	930	345
Total	75	271	12,971	16,447	6334

^a^ Haplogroups are assigned according to the work of [12]. ^b^ Number of mares with breeding values with a reliability ≥ 30%. ^c^ Number of mares varies between traits, as indicated in Table 2. The values shown here are those of the trait “dressage and show jumping competitions of young horses” (ABP) jumping with the highest number of mares.

**Table 2:** Descriptive statistics of the breeding values and reliabilities estimated on behalf of the FN and on behalf of the Holstein Breeding Association for studbook inspection and mare performance test.

		Breeding Value ^a^		Reliability	
Trait ^b^	n	Mean (SD ^c^)	Range	Mean (SD ^c^)	Range
FN genetic evaluation					
TSP jumping	12,656	105.86 (13.95)	48−148	41.82 (5.50)	30−75
TSP dressage	11,045	86.58 (9.18)	56−136	35.96 (3.92)	30−66
ABP jumping	12,971	99.09 (13.87)	31−137	48.75 (7.64)	30−78
ABP dressage	12,231	84.83 (12.00)	34−147	43.41 (7.19)	30−68
ZP walk	12,482	86.55 (10.41)	39−136	44.51 (8.59)	30−71
ZP trot	12,482	82.48 (12.01)	31−141	48.12 (10.82)	30−76
ZP canter	12,724	88.45 (13.04)	29−143	48.04 (10.33)	30−75
ZP rideability	12,701	85.32 (12.65)	34−143	47.15 (9.83)	30−74
ZP free jumping	12,587	104.56 (16.01)	45−149	43.60 (7.01)	30−71
ZP parcours jumping	12,599	102.17 (13.61)	44−142	40.59 (4.59)	30−70
JPf jumping	12,918	101.45 (16.58)	31−148	48.09 (6.83)	30−79
JPf dressage	12,918	82.26 (13.74)	24−149	49.81 (11.06)	30−77
ZP jumping	12,823	103.46 (16.39)	43−149	44.20 (5.95)	30−72
ZP dressage	12,696	82.78 (13.59)	24−149	51.13 (12.38)	30−80
HEK jumping	10,711	109.37 (18.95)	50−178	48.15 (7.96)	30−81
HEK dressage	5302	89.95 (12.35)	60−161	35.03 (4.77)	30−63
Studbook inspection					
HOL type	16,447	95.34 (19.88)	10−169	58.27 (3.83)	30−83
HOL topline	16,447	94.58 (20.97)	5−168	58.27 (3.83)	30−83
HOL forehand	16,447	92.48 (20.85)	4−165	58.27 (3.83)	30−83
HOL hindquarters	16,447	90.82 (22.45)	4−173	58.27 (3.83)	30−83
HOL correctness of gaits	16,447	94.94 (19.68)	11−187	58.27 (3.83)	30−83
HOL impulsion	16,447	95.19 (19.69)	13−189	58.27 (3.83)	30−83
Mare performance test					
HOL walk	6334	98.81 (23.54)	3−214	52.13 (7.83)	30−69
HOL trot	6334	96.83 (22.19)	12−187	52.13 (7.83)	30−69
HOL canter	6334	94.19 (21.91)	4−189	52.13 (7.83)	30−69
HOL rideability	6334	94.59 (22.81)	11−200	52.13 (7.83)	30−69
HOL free jumping	6334	85.10 (22.14)	3−149	52.13 (7.83)	30−69

^a^ Only breeding values with a reliability ≥ 30% are considered. ^b^ TSP = show jumping and dressage competitions, ABP = show jumping and dressage competitions of young horses, ZP = own performance tests of mares and stallions, JPf = sport and breeding tests of young horses, HEK = highest level in competition, HOL = Holstein Breeding Association. ^c^ SD = Standard deviation.

**Table 3:** Overview of mtSNPs used for mitochondrial association analyses and description of associated mtSNPs.

Genetic Data	
Total number of substitutions	467
Non-synonymous mtSNPs	101
Non-synonymous mtSNPs with MAF > 0.01	51
**Results**	
Total number of associated mtSNPs	24
Number of mtSNPs associated with FN-EBVs ^a^	20
Number of mtSNPs associated with HOL-EBVs ^b^	20
Number of mtSNP pairs in total LD (r^2^ = 1)	4
Number of mtSNPs triplets in total LD (r^2^ = 1)	2

^a^ Breeding values estimated by the Fédération Équestre Nationale. ^b^ Breeding values estimated by the Holstein Breeding Association.

## Data Availability

The data set containing the mitochondrial haplotypes analyzed during the current study can be found in the additional supporting files. The phenotypic data that support the findings of this study are available from the Holstein Breeding Association and the Fédération Équestre Nationale, but restrictions apply to the availability of these data, which were used under license for the current study, and so are not publicly available. Data are, however, available from the authors upon reasonable request and with permission of Holstein Breeding Association and the Fédération Équestre Nationale.

## Supplementary Materials

The following supporting information can be downloaded at: https://www.mdpi.com/article/10.3390/ani12060704/s1, File S1: Breeding value estimation for the Holstein horse, File S2: Mitochondrial haplotypes.

## Appendix A

**Table A1:** Results for the association between FN breeding values (EBV) and significant mitochondrial SNPs (p < 5 x 10^−8^), average breeding values for the two alleles, and distribution of the minor allele among haplogroups.

Trait	mtSNP ^a^	n	Alleles ^b^	MAF	Ø EBV Major Allele (SD)	Ø EBV Minor Allele (SD)	Occurrence of Minor Allele in Haplogroups (%) ^c^								*p*-Value
							B	D	G	I	K	L	N	P	
TSP jumping	mtDNA_2216	12,656	C/T	0.023	105.74 (13.92)	111.03 (14.23)		100							1.983 × 10^−10^
	mtDNA_2607	12,656	C/T	0.039	106.01 (13.92)	102.28 (14.24)						18.75			4.293 × 10^−09^
TSP dressage	mtDNA_9963	11,045	G/A	0.224	86.29 (9.11)	87.48 (9.35)	100								7.325 × 10^−09^
ABP jumping	mtDNA_2607	12,971	C/T	0.039	99.25 (13.80)	95.12 (14.76)						18.75			4.380 × 10^−11^
ABP dressage	mtDNA_4599	12,231	C/G	0.124	85.08 (11.96)	83.03 (12.12)							76.92		2.638 × 10^−10^
	mtDNA_8007	12,231	A/G	0.269	84.34 (11.98)	86.17 (11.96)	100	100							3.821 × 10^−14^
	mtDNA_9963	12,231	G/A	0.246	84.34 (11.98)	86.37 (11.92)	100								5.709 × 10^−16^
ZP walk	mtDNA_77	12,482	C/T	0.017	86.63 (10.40)	82.00 (9.77)						6.25			1.620 × 10^−10^
	mtDNA_7600	12,482	G/C	0.018	86.62 (10.39)	82.52 (10.48)	4.17								3.067 × 10^−09^
	mtDNA_8007	12,482	A/G	0.265	86.21 (10.30)	87.50 (10.64)	100	100							9.512 × 10^−10^
	mtDNA_9963	12,482	G/A	0.239	86.19 (10.30)	87.67 (10.67)	100								1.260 × 10^−11^
	mtDNA_15299	12,482	C/T	0.013	86.61 (10.41)	81.84 (9.25)			11.11						4.391 × 10^−09^
ZP trot	mtDNA_77	12,482	C/T	0.017	82.58 (11.99)	77.16 (12.25)						6.25			4.254 × 10^−11^
	mtDNA_1608	12,482	A/T	0.173	82.79 (12.04)	81.01 (11.78)							100		3.203 × 10^−10^
	mtDNA_4599	12,482	C/G	0.125	82.73 (11.98)	80.76 (12.08)							76.92		1.158 × 10^−09^
	mtDNA_8007	12,482	A/G	0.268	82.10 (11.88)	83.56 (12.32)	100	100							1.755 × 10^−09^
	mtDNA_9963	12,482	G/A	0.242	82.07 (11.87)	83.82 (12.37)	100								2.748 × 10^−12^
ZP canter	mtDNA_77	12,724	C/T	0.017	88.59 (12.99)	80.57 (13.89)						6.25			2.200 × 10^−16^
	mtDNA_1608	12,724	A/T	0.169	88.75 (13.09)	87.00 (12.73)							100		1.273 × 10^−08^
	mtDNA_4599	12,724	C/G	0.123	88.73 (13.05)	86.49 (12.83)							76.92		1.994 × 10^−10^
	mtDNA_8007	12,724	A/G	0.263	87.98 (12.98)	89.77 (13.12)	100	100							7.456 × 10^−12^
	mtDNA_9963	12,724	G/A	0.238	87.99 (12.99)	89.92 (13.11)	100								1.260 × 10^−12^
ZP rideability	mtDNA_77	12,701	C/T	0.017	85.45 (12.61)	78.01 (12.60)						6.25			2.200 × 10^−16^
	mtDNA_4599	12,701	C/G	0.123	85.58 (12.63)	83.47 (12.60)							76.92		5.726 × 10^−10^
	mtDNA_4646	12,701	C/T	0.241	84.96 (12.56)	86.48 (12.87)	100								6.596 × 10^−09^
	mtDNA_9963	12,701	G/A	0.238	84.92 (12.57)	86.59 (17.82)	100								2.243 × 10^−10^
ZP free jumping	mtDNA_2607	12,587	C/T	0.039	104.78 (15.87)	99.39 (18.22)						18.75			1.588 × 10^−13^
	mtDNA_4599	12,587	C/G	0.123	104.86 (15.98)	102.42 (16.03)							76.92		1.565 × 10^−08^
ZP parcours jumping	mtDNA_2607	12,599	C/T	0.039	102.34 (13.53)	98.21 (14.79)						18.75			3.446 × 10^−11^
JPf jumping	mtDNA_2607	12,918	C/T	0.039	101.65 (16.48)	96.39 (18.02)						18.75			2.553 × 10^−12^
JPf dressage	mtDNA_77	12,823	C/T	0.017	82.38 (13.71)	75.76 (14.08)						6.25			1.398 × 10^−12^
	mtDNA_4599	12,823	C/G	0.123	82.54 (13.73)	80.27 (13.68)							76.92		7.841 × 10^−10^
	mtDNA_8007	12,823	A/G	0.264	81.76 (13.65)	83.63 (13.91)	100	100							1.348 × 10^−11^
	mtDNA_9963	12,823	G/A	0.238	81.76 (13.65)	83.87 (13.90)	100								1.136 × 10^−13^
ZP jumping	mtDNA_2607	12,696	C/T	0.039	103.67 (16.28)	98.26 (18.27)						18.75			3.863 × 10^−13^
ZP dressage	mtDNA_77	12,801	C/T	0.017	82.91 (13.55)	75.37 (13.86)						6.25			2.900 × 10^−16^
	mtDNA_4599	12,801	C/G	0.123	83.05 (13.59)	80.87 (13.41)							76.92		2.268 × 10^−09^
	mtDNA_8007	12,801	A/G	0.264	82.34 (13.46)	84.03 (13.87)	100	100							5.207 × 10^−10^
	mtDNA_9963	12,801	G/A	0.238	82.32 (13.46)	84.27 (13.89)	100								4.479 × 10^−12^
HEK jumping	mtDNA_1605	10,711	G/A	0.011	109.26 (18.93)	119.26 (18.74)				20.00					6.616 × 10^−09^
	mtDNA_2216	10,711	C/T	0.022	109.28 (18.88)	117.92 (20.22)		100							1.877 × 10^−12^
	mtDNA_14827	10,711	G/A	0.085	109.01 (18.92)	113.25 (18.94)		100	11.11	20.00		37.50	7.69		1.061 × 10^−10^

^a^ The following mtSNPs show perfect LD and only one mtSNP is declared in the subsequent evaluations: mtDNA_9963-mtDNA_1386-mtDNA_10766; mtDNA_1608-mtDNA_2339−mtDNA_4526; mtDNA_2216−mtDNA_11459; mtDNA_1605−mtDNA_13726; mtDNA_8007−mtDNA_11844; mtDNA_4646−mtDNA_13335. ^b^ Major/minor allele. ^c^ Percentage of lineages in the respective haplogroup carrying the minor allele. Haplogroups are assigned according to the work of [12].

**Table A2:** Results for the association between breeding values (EBV) for studbook inspection, mare performance test and significant mitochondrial SNPs (*p* < 5 × 10^−8^), average breeding values for the two alleles, and distribution of the minor allele among haplogroups.

Trait	mtSNP ^a^	n	Alleles ^b^	MAF	Ø EBV Major Allele (SD)	Ø EBV Minor Allele (SD)	Occurrence of Minor Allele in Haplogroups (%) ^c^								*p*-Value
							B	D	G	I	K	L	N	P	
Studbook inspection															
HOL type	mtDNA_77	16,447	C/T	0.019	95.54 (19.88)	85.09 (17.44)						6.25			2.200 × 10^−16^
	mtDNA_1382	16,447	C/T	0.056	95.13 (19.89)	98.90 (19.48)								100	1.977 × 10^−08^
HOL topline	mtDNA_77	16,447	C/T	0.019	94.79 (20.96)	83.36 (18.23)						6.25			2.200 × 10^−16^
	mtDNA_9197	16,447	T/C	0.012	94.46 (20.97)	104.37 (19.26)			11.11						4.124 × 10^−11^
HOL forehand	mtDNA_77	16,447	C/T	0.019	92.66 (20.86)	83.18 (18.26)						6.25			1.688 × 10^−15^
	mtDNA_7600	16,447	G/C	0.017	92.62 (20.16)	84.60 (22.16)	4.17								1.774 × 10^−10^
	mtDNA_9774	16,447	G/A	0.011	92.58 (20.76)	83.67 (26.86)	4.17								2.089 × 10^−08^
HOL hindquarters	mtDNA_1382	16,447	C/T	0.056	90.51 (22.42)	96.02 (22.31)								100	3.562 × 10^−13^
	mtDNA_7600	16,447	G/C	0.017	90.95 (22.44)	83.46 (21.91)	4.17								3.122 × 10^−08^
	mtDNA_9197	16,447	T/C	0.012	90.69 (22.45)	101.64 (20.11)			11.11						1.004 × 10^−11^
	mtDNA_14653	16,447	A/G	0.021	90.99 (22.46)	83.01 (20.67)			33.33						6.133 × 10^−11^
HOL correctness of gaits	mtDNA_77	16,447	C/T	0.019	95.13 (19.66)	85.01 (18.09)						6.25			2.200 × 10^−16^
	mtDNA_7600	16,447	G/C	0.017	95.07 (19.66)	87.40 (19.30)	4.17								9.645 × 10^−11^
	mtDNA_14653	16,447	A/G	0.021	95.09 (19.69)	87.79 (17.72)			33.33						8.903 × 10^−12^
HOL impulsion	mtDNA_77	16,447	C/T	0.019	95.34 (19.76)	87.12 (19.06)						6.25			2.666 × 10^−13^
	mtDNA_1382	16,447	C/T	0.056	94.95 (19.74)	99.15 (18.52)								100	2.482 × 10^−10^
	mtDNA_7600	16,447	G/C	0.017	95.31 (19.68)	87.83 (18.92)	4.17								2.829 × 10^−10^
	mtDNA_9774	16,447	G/A	0.011	95.31 (19.62)	84.03 (23.45)	4.17								5.447 × 10^−14^
	mtDNA_14653	16,447	A/G	0.021	95.37 (19.69)	86.86 (17.61)			33.33						1.897 × 10^−15^
Mare performance test															
HOL canter	mtDNA_77	6334	C/T	0.016	94.41 (20.08)	81.09 (20.55)						6.25			9.199 × 10^−10^
	mtDNA_1608	6334	A/T	0.160	94.99 (22.23)	89.97 (21.25)							100		2.009 × 10^−11^
	mtDNA_4599	6334	C/G	0.151	91.24 (22.12)	85.94 (21.73)							76.92		2.666 × 10^−10^
	mtDNA_4646	6334	C/T	0.258	93.12 (22.05)	97.24 (22.17)	100								5.784 × 10^−11^
	mtDNA_8007	6334	A/G	0.277	92.99 (22.06)	97.31 (22.09)	100	100							2.000 × 10^−12^
HOL walk	mtDNA_4646	6334	C/T	0.258	97.84 (22.54)	101.61 (24.12)	100								2.351 × 10^−08^
	mtDNA_15299	6334	C/T	0.012	99.01 (22.99)	82.84 (17.19)			11.11						1.249 × 10^−09^
HOL rideability	mtDNA_77	6334	C/T	0.016	94.85 (22.27)	78.25 (21.13)						6.25			2.088 × 10^−13^
	mtDNA_1608	6334	A/T	0.160	95.31 (22.34)	90.79 (22.19)							100		6.809 × 10^−09^
	mtDNA_4599	6334	C/G	0.151	95.21 (22.23)	89.88 (22.85)							76.92		4.052 × 10^−12^
	mtDNA_4646	6334	C/T	0.258	93.52 (22.20)	97.64 (22.64)	100								3.055 × 10^−10^
	mtDNA_8007	6334	A/G	0.277	93.50 (22.18)	97.42 (22.67)	100	100							8.262 × 10^−10^
	mtDNA_14653	6334	A/G	0.021	94.83 (22.38)	82.56 (19.07)			33.33						1.183 × 10^−09^

^a^ The following mtSNPs show perfect LD and only one mtSNP is declared in the subsequent evaluations: mtDNA_9963-mtDNA_1386-mtDNA_10766; mtDNA_1608-mtDNA_2339−mtDNA_4526; mtDNA_4646−mtDNA_13335. ^b^ Major/minor allele. ^c^ Percentage of lineages in the respective haplogroup carrying the minor allele. Haplogroups are assigned according to the work of [12].
