# Supplementary material for: Mitochondrial DNA Variation Contributes to the Aptitude for Dressage and Show Jumping Ability in the Holstein Horse Breed
Source: Animals (Basel). 2022 Mar 11;12(6):704. doi: 10.3390/ani12060704 (PMC8944467; doi:10.3390/ani12060704)
Supplement: Supplementary file 1 [file animals-12-00704-s001.zip › Supplementary File S1.pdf]

Supplementary File S1

Breeding value estimation for the Holstein horse

For the Holstein horse, breeding values (EBVs) are estimated on behalf of both, the Holstein Breeding Association (HOL) and the Fédération Équestre Nationale (FN). The HOL runs three different genetic evaluations based on the results from foal registration, studbook inspection (SBI) and mare performance test (MPT) for the Holstein population. During SBI the type, topline, forehand, hindquarters and the basic gaits (walk, trot and canter) are assessed in free movement. A considerably smaller number of mares used for breeding also completes MPT, either as one-day field or 14-day station test. During both tests, basic gaits under the rider, the rideability and free jumping are judged. On the national level, EBVs from the integrated genetic evaluation on behalf of the FN are available for all warmblood horse breeding associations in Germany, providing a summarized genetic assessment of all traits generated from equestrian sport events as well as broodmare and stallion performance tests. There are different sources of information: Evaluations are based on (i) results of show jumping and dressage competitions (TSP), for which the transformed rank within the competition is used, (ii) results of sport and breeding tests for young horses (JPf), namely the score achieved in own performance tests of mares or stallions (ZP) and show jumping and dressage competitions of young horses (ABP), and (iii) the newly introduced trait "highest level in competition (HEK)", which is not only based on national TSP data but also on international competition results from German horses provided by the Fédération Équestre Internationale (FEI). The classification of HEK is made according to the highest performance class (ranging from A to S****) and the achieved rank with differentiation between participation, 2nd to 4th rank and 1st rank [8].

The FN estimates a total of 12 different EBVs for the traits TSP jumping, TSP dressage, ABP jumping, ABP dressage, ZP walk, ZP trot, ZP canter, ZP rideability, ZP free jumping, ZP parcours jumping, HEK jumping and HEK dressage. Another four EBVs (JPf jumping, JPf dressage, ZP

jumping and ZP dressage) are calculated from the previous ones as indicated in Table S1 [2]. For the estimation a best linear unbiased prediction (BLUP) multi-trait repeatability animal model is applied except for the trait HEK, a comprehensive performance trait for equine sport, for which a BLUP animal model is used. Each trait is corrected for its own relevant environmental effects. Each single performance of a horse is directly compared with the performance of horses that started in the same competition or the yearly test group of a test station. For sport events, the effects of age * sex and the rider's competition groups are considered. Riders having many observations on different horses are considered as a separate fixed effect. For ZP, the model is reduced to the fixed effect of the respective yearly test group. The HEK traits are adjusted for the fixed effects of sex, the year of achieving the highest level and the age of the horse at its latest start [9].

Table S1. Weights for the combined breeding values JPf jumping, JPf dressage, ZP jumping and ZP dressage according to [9].

	JPf jumping	JPf dressage	ZP jumping	ZP dressage
ABP jumping	0.5			
ABP dressage		0.5		
ZP walk		0.625		0.25
ZP trot		0.625		0.25
ZP canter		0.625		0.25
ZP rideability		0.625		0.25
ZP free jumping	0.25		0.5	
ZP parcours jumping	0.25		0.5	

The HOL-EBVs are estimated using a BLUP multi-trait animal model. The evaluation for SBI includes the six traits HOL type, HOL topline, HOL forehand, HOL hindquarters, HOL correctness of gaits and HOL impulsion. HOL correctness of gaits and HOL impulsion are estimated based on the scores for trot and canter, respectively. The following fixed effects are considered for SBI: inspection year, season of inspection (four classes: January – March, June, July – August, September – December), the breeding district of the horse owner (n = 12) and age group (five classes: 2.00 – 3.24 years, 3.25 – 3.74 years, 3.75 – 4.24 years, 4.25 – 7.24 years, ≥ 7.25 years). The evaluation for

MPT includes the five traits HOL walk, HOL trot, HOL canter, HOL rideability and HOL free jumping and considers the effect of test group * test year * test type.

EBVs for SBI and MPT were estimated for a total of 61,022 and 27,041 individuals in the pedigree, respectively. Information on performance for SBI and MPT was available for 57,996 and 11,451 mares, respectively. For SBI and MPT, 30.60 % and 71.32 % of the mares with performance information belonged to the 75 lineages whose mtDNA was sequenced in [5] and that are considered in our study for the mitochondrial association analysis. FN-EBVs were estimated for 821,198 horses based on 23,828,781 results from competitions and breeding tests for 591,564 horses.
